# Personal

**Published:** 1907-07

**Authors:** 


### PERSONAL.

Dr. James Wright Putnam, of Buffalo, one of the associate editors of the Journal, and a former president of the American Neurological Association, will sail for Europe, July 5, on the Red Star liner Menomene, from Philadelphia. Dr. Putnam will attend the International Congress of Neurology, Psychology and Psychiatry at Amsterdam, September 2-7, 1907, having been specially invited by the president of the congress.

Dr. Ida C. Bender, of Buffalo, will attend the international congress of school hygiene, to be held in London, August 5-10, 1907, as Buffalo's official representative. The education department of Buffalo was officially invited to send a representative by Sir Lauder Brunton, the president of the congress, and Dr. Bender was chosen as the official delegate. She will endeavor to have the congress hold its next session in Buffalo.

Dr. Edwin Walker, of Evansville, Ind., was elected first vice-president of the American Medical Association at the annual meeting held at Atlantic City in June, 1907. Dr. Walker is one of the most prominent physicians in the southwest, and has been an active scientific worker in the association for many years.

Dr. Walter Blackburn Dorsett, of Saint Louis, was elected chairman of the section on obstetrics and gynecology of the American Medical Association during its recent meeting held at Atlantic City. This is a deserved recognition of able and efficient service rendered the section by Dr. Dorsett for many years.

Dr. C. W. Bourne, of Hamburg, was elected medical director of the Grand Army of the Republic, department of New York, at the recent annual encampment at Utica. Dr. Bourne served as a surgeon of a Vermont regiment during the civil war and is a companion of the Military Order of the Loyal Legion.

Dr. Joseph Decatur Bryant, of New York, recently received the degree of Doctor of Laws from the New York University. Dr. Bryant is the reigning president of the American Medical Association having taken office at the recent meeting of that body held at Atlantic City.

Dr. William Seaman Bainbridge, of New York, received the degree of Doctor of Science at the annual commencement of the Western University of Pennsylvania, June 7.

Dr. Charles F. Howard, of Buffalo, has been nominated by Governor Hughes to be a member of the prisons commission created by a law recently enacted. His term of service is four years.

---

Dr. W. J. O'Donnell, of Buffalo, received the degree of M.Sc. from St. Bonaventure's college and seminary at its forty-seventh annual commencement held in June, 1907.

---

Dr. Augustus Grote Pohlman, of Bloomington, Ind., has recently been appointed junior professor of anatomy at Indiana University. Dr. Pohlman is a graduate of the University of Buffalo, class of 1900.

